# Fc gamma receptor IIb in tumor-associated macrophages and dendritic cells drives poor prognosis of recurrent glioblastoma through immune-associated signaling pathways

**DOI:** 10.3389/fgene.2022.1046008

**Published:** 2023-01-06

**Authors:** Xiong Jin, Jianlei Kang, Qing Lu, Shuang-Lei Guo, Meichen Liu, Yue Zhang, Can Cui, Hong-Lin Liu, Xin Xu, Jinlong Yin

**Affiliations:** ^1^ Henan Key Laboratory of Brain Targeted Bio-nanomedicine, School of Life Sciences & School of Pharmacy, Henan University, Kaifeng, China; ^2^ Department of Neurosurgery, Affiliated Cancer Hospital of Zhengzhou University & Henan Cancer Hospital, Zhengzhou, China; ^3^ Plastic Surgery Department of the First Affiliated Hospital of Henan University, Kaifeng, China; ^4^ Department of Neurosurgery, Huaihe Hospital of Henan University, Kaifeng, China

**Keywords:** recurrent glioblastoma, Fcgr2b, tumor-associated macrophage, dendritic cell, immune-associated signaling pathway

## Abstract

**Background:** Among central nervous system tumors, glioblastoma (GBM) is considered to be the most destructive malignancy. Recurrence is one of the most fatal aspects of GBM. However, the driver molecules that trigger GBM recurrence are currently unclear.

**Methods:** The mRNA expression data and clinical information of GBM and normal tissues were collected from the Chinese Glioma Genome Atlas The Cancer Genome Atlas (TCGA), and REpository for Molecular BRAin Neoplasia DaTa (REMBRANDT) cohorts. The DESeq2 R package was used to identify the differentially expressed genes between primary and recurrent GBM. ClueGO, Kyoto Encyclopedia of Genes and Genomes (KEGG), Biological Process in Gene ontology (GO-BP), and the Protein ANalysis THrough Evolutionary Relationships (PANTHER) pathway analyses were performed to explore the enriched signaling pathways in upregulated DEGs in recurrent GBM. A gene list that contained potential oncogenes that showed a significant negative correlation with patient survival from The Cancer Genome Atlas was used to further screen driver candidates for recurrent GBM. Univariate Cox proportional hazards regression analyses were used to investigate the risk score for the mRNA expression of the candidates. Single-cell RNA sequencing (scRNA-Seq) analyses were used to determine the cell type-specific distribution of Fc gamma receptor II b (FcγRIIb) in GBM. Immunohistochemistry (IHC) was used to confirm the FcγRIIb-positive cell populations in primary and paired recurrent GBM.

**Results:** Through DEG analysis and overlap analysis, a total of 10 genes that are upregulated in recurrent GBM were screened. Using validation databases, FcγRIIb was identified from the 10 candidates that may serve as a driver for recurrent GBM. *FCGR2B* expression, not mutation, further showed a highly negative correlation with the poor prognosis of patients with recurrent GBM. Furthermore, scRNA-Seq analyses revealed that tumor-associated macrophage- and dendritic cell-specific FCGR2B was expressed. Moreover, FcγRIIb also showed a strong positive correlation coefficient with major immune-associated signaling pathways. In clinical specimens, FcγRIIb-positive cell populations were higher in recurrent GBM than in primary GBM.

**Conclusion:** This study provides novel insights into the role of FcγRIIb in recurrent GBM and a promising strategy for treatment as an immune therapeutic target.

## Introduction

Glioblastoma (GBM) is the most aggressive and incurable malignancy in the central nervous system. Despite continuous encouraging developments in cancer therapy in recent decades, the median survival time is still less than 15 months (Sung et al., 2021). One of the major issues for GBM treatment is recurrence. Bevacizumab (also known as Avastin) is the only effective therapeutic drug approved by the Food and Drug Administration (FDA) for the treatment of recurrent GBM (Wick et al., 2017). Recent studies have mainly focused on combined therapies with Avastin and radiation and/or chemical drugs; nevertheless, the results for the improvement of the survival rate of GBM patients are still not optimistic (Ren et al., 2021). Consequently, finding a promising therapeutic strategy and providing more efficient treatment options to patients with recurrent GBM have become the primary goals of oncologists.

Fragment crystallizable receptors (FcRs) are immune cell-expressing proteins that bind to the Fc region of immunoglobulin (IgG). FcRs play critical roles in modulating the crosstalk between innate and adaptive immune responses. Fc gamma receptor II b (FcγRIIb, coded by *FCGR2B*), also known as CD32a, is a member of the FcR family that regulates multiple inflammatory and immune responses by inhibiting cytokines release (Smith and Clatworthy, 2010). Among the FcR family members, FcγRIIb acts as the only inhibitory FcR that inhibits B-cell activation, while FcγRI (CD64) shows a high affinity, and FcγRIIa (CD32a), FcγRIIc (CD32c), FcγRIIIa (CD16a), and FcγRIIIb (CD16b) show a low affinity of interacting with several immunoglobulin (IgG)-type antibodies (Nimmerjahn and Ravetch, 2008). The I232T (isoleucine changed to threonine at position 232) missense mutation on *FCGR2B* caused by a single-nucleotide polymorphism is associated with susceptibility to autoimmune diseases such as systemic lupus erythematosus (Willcocks et al., 2010). Based on the cell types expressed and their roles in immune responses, many efforts have been made to clarify the therapeutic efficacy of targeting FcγRIIb in autoimmune diseases and hematopoietic cancers (Nakamura et al., 2000; Boross et al., 2011; Li and Ravetch, 2011; Roghanian et al., 2015). However, the role of FcγRIIb in regulating the invasiveness of GBM, especially recurrent GBM, is unknown.

Here, we show that various immune-related signaling pathways are enriched in recurrent GBM using an RNA-Seq database from the Chinese Glioma Genome Atlas (CGGA). Through a differentially expressed gene (DEG) analysis, we found 10 candidates that were upregulated in recurrent GBM. In particular, only FcγRIIb is expressed at a higher mRNA level in GBM than in normal or low-grade glioma tissues and shows a negative correlation with the survival time of GBM patients, especially of recurrent GBM patients, from multiple glioma databases, including The Cancer Genome Atlas (TCGA) and REpository for Molecular BRAin Neoplasia DaTa (REMBRANDT). Furthermore, in a single-cell RNA-Seq (scRNA-Seq) analysis, we observed that *FCGR2B* is mainly expressed in tumor-associated macrophages (TAMs) and dendritic cells (DCs) and is preferentially expressed in recurrent GBM. Moreover, we identified that multiple immune-associated signaling pathways regulated by FcγRIIb in recurrent GBM *via* gene set enrichment analysis (GSEA). We further confirmed that higher FcγRIIb-positive cells in recurrent GBM compared to primary GBM. Our findings characterize FcγRIIb in recurrent GBM and suggest a promising therapeutic strategy for FcγRIIb in the treatment of recurrent GBM.

## Data and methods

### Public data collection

We collected RNA sequencing (RNA-Seq, batch 1) data and clinical information from the Chinese Glioma Cancer Atlas (CGGA) (Zhao et al., 2021) and mRNA microarray data and clinical information of the REpository for Molecular BRAin Neoplasia DaTa (REMBRANDT) (Madhavan et al., 2009) from the CGGA portal (http://www.cgga.org.cn/), RNA-Seq data and clinical information of The Cancer Genome Atlas (TCGA) (Brennan et al., 2013) from the UCSC xena portal (https://xena.ucsc.edu/public).

### Differentially expressed gene (DEG) analysis

DEG analysis between primary and recurrent GBM was performed using the DESeq2 R package (Love et al., 2014). Genes with Benjamini and Hochberg’s adjusted *p* < 0.05 as determined by DESeq2 were considered differentially expressed. The resulting DEG graphs are presented as a volcano plot constructed using the ggplot2 R package. Kyoto Encyclopedia of Genes and Genomes (KEGG) pathway (Kanehisa and Goto, 2000), the biological process of gene ontology (Ashburner et al., 2000) and ClueGO (Bindea et al., 2009) were used to identify enriched signaling pathways. The resulting graphs are presented as matrix bubble charts constructed using the ggbubbles R package.

### Patient survival analysis

All patient survival analyses were performed using the Kaplan‒Meier estimate, and the differences between the two groups were measured using the log-rank (Mantel‒Cox) test. The hazard ratio (HR) was calculated using Cox proportional hazards regression analysis. A *p*-value less than 0.05 was considered significantly different. Overall survival was used as the primary endpoint. For patients who were alive at the time of the last follow-up, survival records were censored from the analysis. GraphPad PRISM software was used to construct the survival Kaplan‒Meier plot and conduct the statistical analyses.

### Pan-cancer analysis

Transcript expression in normal and tumor tissues and univariate Cox regression HR of *FCGR2B* in pan-cancer from the TCGA cohort were analyzed in HOME for researchers. Expression is shown as transcripts per million (TPM), and the resulting graphs are presented as dot plots. Higher expression in normal samples is presented as green labels, while higher expression in tumor samples is presented as red labels. A *p*-value less than 0.05 was considered significantly different.

### Single cell RNA sequencing (scRNA-Seq) analysis

For Figure 5A, the scRNA-Seq gene expression profile was collected from the Brain Tumor Immune Micro Environment (Brain TIME) portal (https://joycelab.shinyapps.io/braintime/) (Klemm et al., 2020). GraphPad PRISM software was used to construct the dot plots and conduct the statistical analyses. For Figure 5B, the scRNA-Seq gene expression of GSE131928 (Neftel et al., 2019) was analyzed, and the t-distributed stochastic neighbor embedding (t-SNE) graph was constructed in the Single Cell Portal from the Broad Institute (https://singlecell.broadinstitute.org/single_cell). For Figures 5C, D, the scRNA-Seq gene expression of GSE163120 (Pombo Antunes et al., 2021) was analyzed, and Uniform Manifold Approximation and Projection (UMAP) graphs were constructed in the Brain Immune Atlas (www.brainimmuneatlas.org).

### Correlation analysis

Pearson’s correlation coefficient between *FCGR2B* and all the other genes in the TCGA GBM RNA-Seq cohort was analyzed in the LinkedOmics portal (linkedomics.org) (Vasaikar et al., 2018) and the resulting graphs are presented as a volcano plot and heatmaps. Genes with R values greater than 0.00 and *p* values less than 0.05 were considered positively correlated significant genes, while genes with R values less than 0.00 and *p* values less than 0.05 were considered significantly negatively correlated genes. Pearson’s correlation coefficient between the mRNA expression of *FCGR2B* and the enrichment score of immune-associated signaling pathways in the CCGA recurrent GBM RNA-Seq cohort was analyzed and constructed using the corrplot R package.

### Gene set enrichment analysis (GSEA)

GSEA (Subramanian et al., 2005) was applied to identify the enriched Protein ANalysis THrough Evolutionary Relationships (PANTHER) pathway (Mi and Thomas, 2009) in the LinkedOmics portal. The resulting graphs are presented as a bar chart and a GSEA chart. The bar graph shows the top 10 enriched signaling pathways. Single sample gene set enrichment analysis (ssGSEA) for the enrichment score of immune-associated signaling pathways in the *FCGR2B* high group from the TCGA cohort was analyzed using the ssGSEA module in the GenePattern portal (Reich et al., 2006).

### Immunohistochemistry (IHC)

Paraffin sections were incubated with citrate buffer (pH 6.0) for antigen retrieval and were incubated with 3% hydrogen peroxide for endogenous peroxidase blocking. Then, paraffin sections were incubated with FcγRIIb antibody (1:50, Sangon Biotech) overnight at 4 C˚ in a humidified chamber and were developed with 3,3′-diaminobenzidine (DAB; Vector Laboratories) for DAB staining.

## Results

### Identification of differentially enriched signaling pathways from recurrent glioblastoma

**FIGURE 1:**
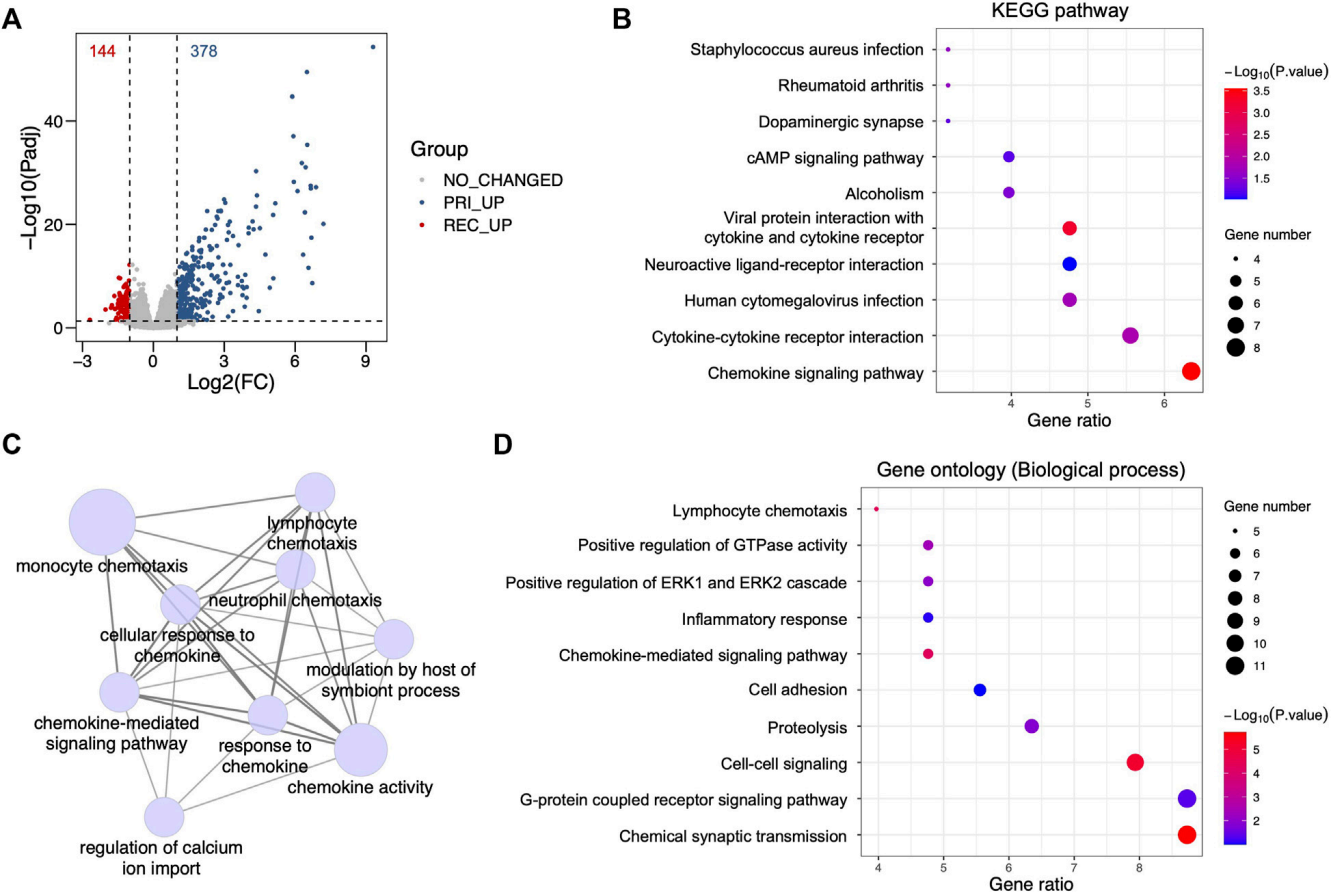
Identification of differentially enriched signaling pathways from recurrent glioblastoma. **(A)** Volcano plot showing differentially expressed genes between primary and recurrent glioblastoma (GBM) from the Chinese Glioma Genome Atlas (CGGA) cohort using a criterion with fold change (FC) and adjacent *p*-value (Padj). Red dots, upregulated genes in recurrent GBM, Log_2_(FC) < -1 and Padj <0.05; gray dots, genes showing no changed FC and no significant *p*-value, |Log_2_(FC)| < 1 and Padj >0.05; blue dots, upregulated genes in primary GBM, Log_2_(FC) > 1 and Padj <0.05. **(B)** Bubble plot for the Kyoto Encyclopedia of Genes and Genomes (KEGG) pathway analysis with upregulated genes in recurrent GBM from the CGGA cohort. **(C)** Signaling network showing the ClueGO analysis for the biological process of gene ontology (GO-BP) with upregulated genes in recurrent GBM from the CGGA cohort. **(D)** Bubble plot showing the GO-BP terms with upregulated genes in recurrent GBM from the CGGA cohort.

To identify the principal regulators of recurrent glioblastoma (GBM), a DEG analysis between primary and recurrent GBM was performed using a glioma database from the Chinese Glioma Genome Atlas (CGGA). We identified 144 and 378 genes that were significantly upregulated in recurrent and primary GBM, respectively (Figure 1A, Supplementary Table S1). With the 144 upregulated genes in recurrent GBM, we performed a Kyoto Encyclopedia of Genes and Genomes (KEGG) pathway analysis and found that various immune-associated signaling pathways were enriched in recurrent GBM, such as the chemokine signaling pathway, cytokine‒cytokine receptor interaction, human cytomegalovirus infection, viral protein interaction with cytokine and cytokine receptor, and *Staphylococcus aureus* infection (Figure 1B). Subsequently, the analyses for the biological process of gene ontology (GO-BP) further demonstrated the crosstalk and high enrichment of multiple immune-associated signaling pathways (Figures 1C, D). Taken together, these results indicate that multiple enriched immune-associated signaling pathways are involved in the recurrence of GBM.

### Identification of differentially expressed genes from recurrent GBM

**FIGURE 2:**
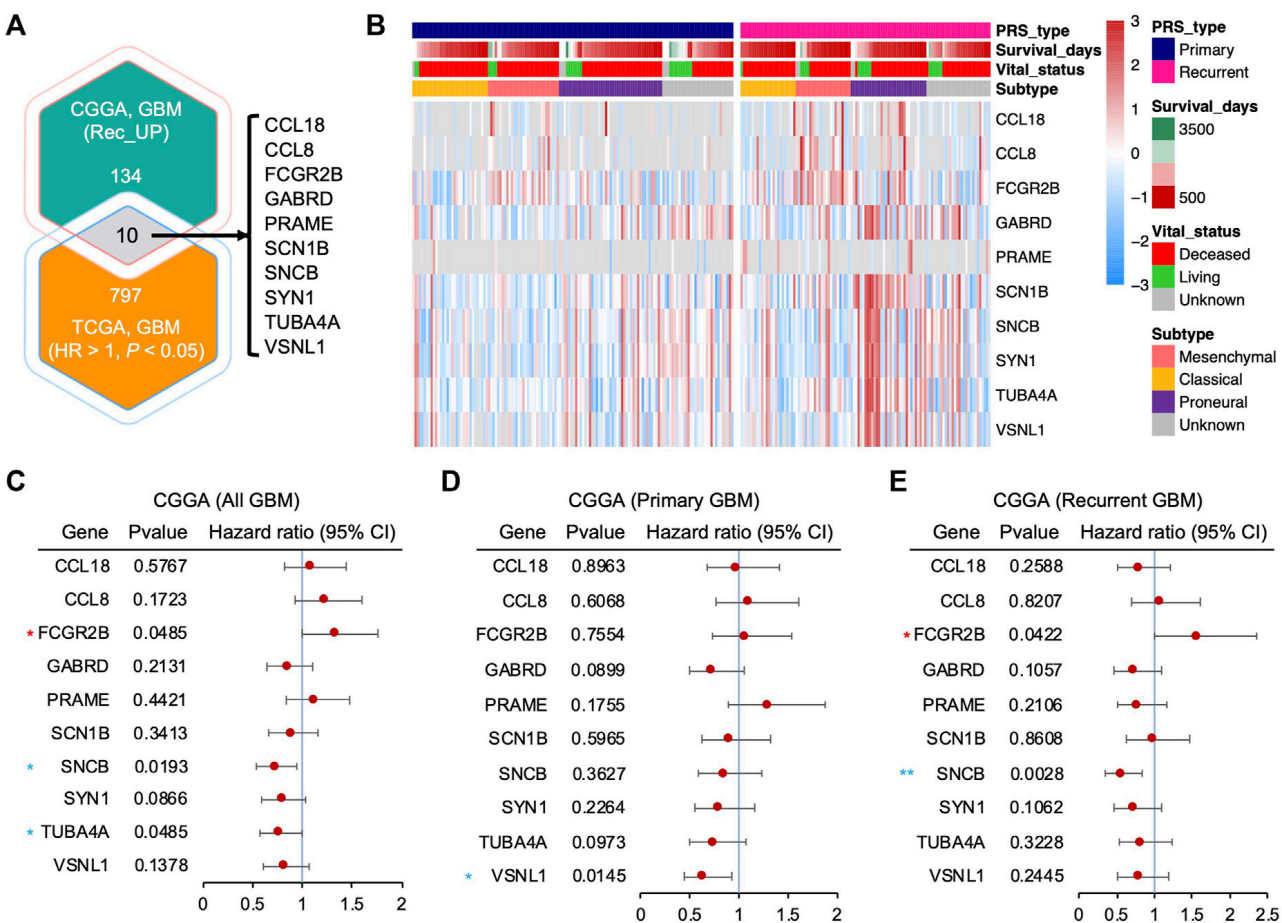
Identification of differentially expressed genes from recurrent GBM. **(A)** Venn diagram showing the intersected gene list between the upregulated genes in recurrent GBM from the CGGA cohort and potential oncogenes from The Cancer Genome Atlas (TCGA) cohort (hazard ratio (HR) > 1 and *p* < 0.05). **(B)** Heatmap depicting the mRNA expression of the potential oncogenic regulator candidates in **(A)** from the CGGA cohort. **(C**–**E)** Forest plots showing the HR of the upregulated genes in all GBMs, including both primary and recurrent GBM **(C)**, primary GBM **(D)**, and recurrent GBM **(E)**, from the CCGA cohort through univariate Cox regression analysis. Horizontal lines represent 95% confidence intervals (CI) of HR. Red asterisk, HR > 1 and *p* < 0.05; blue asterisk, HR < 1 and *p* < 0.05. *p < 0.05, **p < 0.01.

Additionally, to explore the key modulators, we screened candidates with upregulated genes in recurrent GBM from the CGGA cohort and a potential oncogene list (Supplementary Table S2) from The Cancer Genome Atlas (TCGA, RNA-Seq) cohort that contained genes that showed a significantly negative correlation with GBM patient survival (hazard ratio (HR) > 1, *p* < 0.05). A total of 10 genes were identified as potential oncogenic regulators in recurrent GBM, including *CCL18*, *CCL8*, *FCGR2B*, *GABRD*, *PRAME*, *SCN1B*, *SNCB*, *SYN1*, *TUBA4A*, and *VSNL1* (Figure 2A). The heatmap showed a higher mRNA expression of these genes in recurrent GBM than in primary GBM (Figure 2B). Furthermore, a univariate analysis of the potential oncogenic regulators in recurrent GBM using the CGGA cohort was performed. An HR value above 1 is more hazardous for higher mRNA expression levels. For all patient samples with both primary and recurrent GBM, only *FCGR2B* (HR: 1.3220, 95% CI: 0.9988–1.7500) showed a significantly high HR, while *SNCB* (HR: 0.7181, 95% CI: 0.5422–0.9511) and *TUBA4A* (HR: 0.7558, 95% CI: 0.5712–1.0000) showed a remarkably low HR (Figure 2C). Viewed separately, no genes showed significant differences except *VSNL1* (HR: 0.6313, 95% CI: 0.4324–0.9216) in primary GBM (Figure 2D), while *FCGR2B* and *SNCB* were consistent with the results of all GBM samples in recurrent GBM (Figure 2E). Collectively, based on the lack of a correlation between mRNA expression and patient prognosis in primary GBM and a significant association in recurrent GBM, *FCGR2B* is a unique candidate from the list of potential oncogenic regulators that play a fundamental role in recurrent GBM.

### Validation of potential oncogenic regulators from recurrent GBM in other glioma cohorts

**FIGURE 3:**
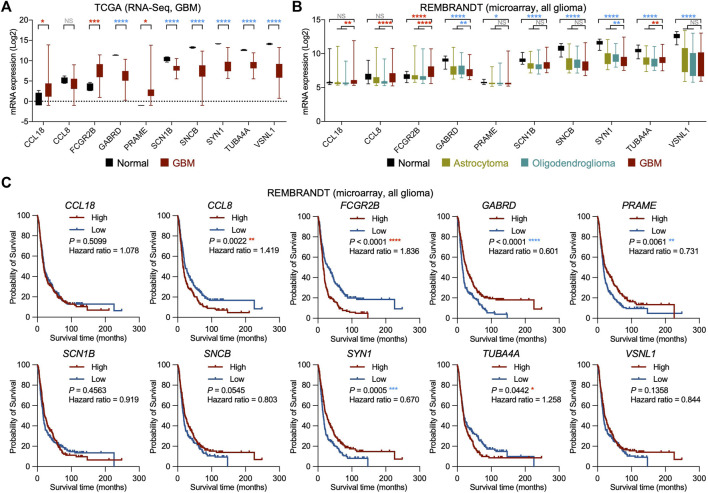
Validation of potential oncogenic regulators from recurrent GBM in other glioma cohorts. **(A)** Box plots showing the mRNA expression of the potential oncogenic regulator candidates in normal and GBM tissues from the TCGA cohort. Red asterisk, significantly higher in GBM; blue asterisk, significantly higher in normal. NS, not significant; *p < 0.05, ***p < 0.001, ****p < 0.0001. **(B)** Box plots showing the mRNA expression of the potential oncogenic regulator candidates in normal, astrocytoma, oligodendroglioma, and GBM tissues from the Repository of Molecular Brain Neoplasia Data (REMBRANDT) cohort. Red asterisk, significantly higher in GBM; blue asterisk, significantly higher in normal or low-grade glioma (LGG, including astrocytoma and oligodendroglioma). NS, not significant; *p < 0.05, **p < 0.01, ****p < 0.0001. **(C)** Kaplan‒Meier survival analyses for all glioma patients with high and low mRNA expression of the potential oncogenic regulator candidates from the REMBRANDT cohort (n = 214, grouped by median value). Red asterisk, worse prognosis in high expression group; blue asterisk, worse prognosis in low expression group. *p < 0.05, **p < 0.01, ***p < 0.001, ****p < 0.0001.

To further investigate our candidates from recurrent glioblastoma, we analyzed the mRNA expression of GBM and normal tissues with the RNA-Seq database from TCGA. Although all of the candidates were classified as potential oncogenic regulators due to the negative correlation between their mRNA expression and patient survival in TCGA, the mRNA expression in GBM was not higher than that in normal tissues for all mRNA. Our analysis showed that the mRNA expression of *CCL18*, *FCGR2B*, and *PRAME* in GBM was significantly higher, and *GABRD*, *SCN1B*, *SNCB*, *SYN1*, *TUBA4A*, and *VSNL1* in GBM were dramatically lower than those in normal tissues, while *CCL8* did not show a significant difference between GBM and normal tissues (Figure 3A). We then compared the mRNA expression of normal tissues, low-grade gliomas (LGGs), including astrocytoma and oligodendroglioma, and GBM using the REpository for Molecular BRAin Neoplasia DaTa (REMBRANDT) cohort. Consistent with the TCGA analysis results, the mRNA expression level of *FCGR2B* was higher in GBM than in normal tissues or LGG (Figure 3B). Similarly, the transcript levels of *GABRD*, *SCN1B*, *SNCB*, *SYN1*, *TUBA4A*, and *VSNL1* were downregulated in GBM compared with normal tissues. However, the expression of *CCL18*, *CCL8*, and *PRAME* in GBM and normal tissues showed differences compared with those in Figure 3A. Next, we measured the correlations between the transcript levels of these candidates and the survival times of glioma patients in the REMBRANDT cohort. Among the 10 candidates, high mRNA expression of *CCL8*, *FCGR2B*, and *TUBA4A* and low mRNA expression of *GABRD*, *PRAME*, and *SYN1* were significantly correlated with worse prognosis, while the mRNA expression of *CCL18*, *SCN1B*, *SNCB*, and *VSNL1* showed no connection to the overall survival of glioma patients (Figure 3C). Taken together, these results support the possibility of FcγRIIb as a potential oncogenic regulator for recurrent GBM.

### FcγRIIb expression is strongly associated with GBM

**FIGURE 4:**
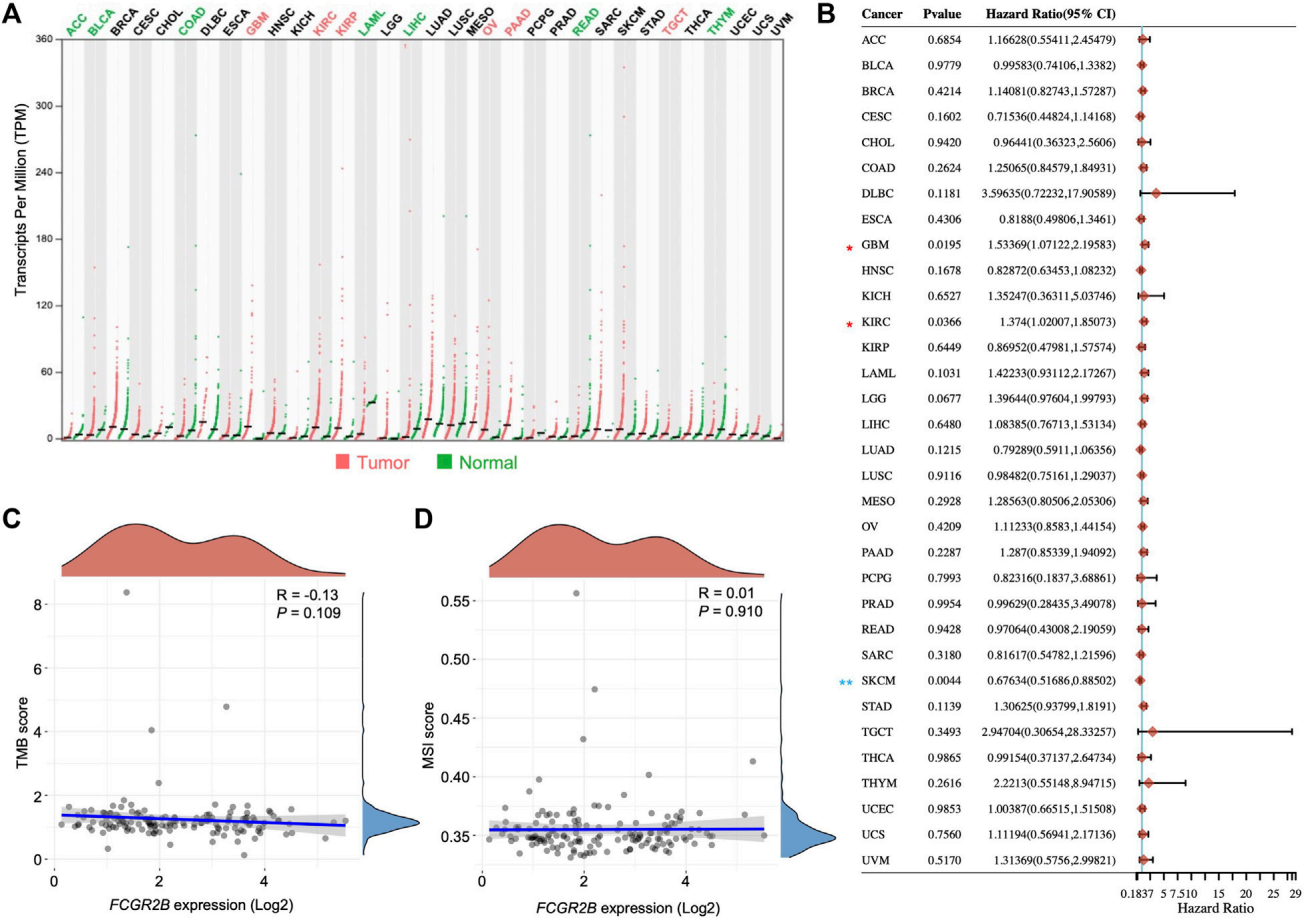
FcγRIIb expression is strongly associated with GBM. **(A)** Dot plots showing the mRNA expression of *FCGR2B* in different tumor types and normal tissues from the TCGA cohort. Red label, significantly higher in tumor; green label, significantly higher in normal; black label, not significant. ACC, adrenocortical carcinoma; BLCA, bladder urothelial carcinoma; BRCA, breast invasive carcinoma; CESC, cervical squamous cell carcinoma and endocervical adenocarcinoma; CHOL, cholangiocarcinoma; COAD, colon adenocarcinoma; DLBC, lymphoid neoplasm diffuse large B-cell lymphoma; ESCA, esophageal carcinoma; HNSC, head and neck squamous cell carcinoma; KICH, kidney chromophobe; KIRC, kidney renal clear cell carcinoma; KIRP, kidney renal papillary cell carcinoma; LAML, acute myeloid leukemia; LIHC, liver hepatocellular carcinoma; LUAD, lung adenocarcinoma; LUSC, lung squamous cell carcinoma; MESO, mesothelioma; OV, ovarian serous cystadenocarcinoma; PAAD, pancreatic adenocarcinoma; PCPG, pheochromocytoma and paraganglioma; PRAD, prostate adenocarcinoma; READ, rectum adenocarcinoma; SARC, sarcoma; SKCM, skin cutaneous melanoma; STAD, stomach adenocarcinoma; TGCT, testicular germ cell tumors; THCA, thyroid carcinoma; THYM, thymoma; UCEC, uterine corpus endometrial carcinoma. **(B)** Forest plots showing the HR of *FCGR2B* in different tumor types from the TCGA cohort through univariate Cox regression analysis. Horizontal lines represent the 95% CI of HR. Red asterisk, HR > 1 and *p* < 0.05; blue asterisk, HR < 1 and *p* < 0.05. *p < 0.05, **p < 0.01. **(C**,**D)** Dot plots depicting the correlation between the mRNA expression of *FCGR2B* and tumor mutational burden (TMB) and microsatellite instability (MSI).

To identify the role of FcγRIIb in cancer and the correlations with the prognosis of patients, a pan-cancer analysis was performed using the TCGA cohort. Through the analysis, we found that the transcripts per million (TPM) of *FCGR2B* were significantly higher in tumor samples than in normal tissues in GBM (fold change of tumor *versus* normal (FC), 53.19), kidney renal clear cell carcinoma (KIRC; FC, 4.67), kidney renal papillary cell carcinoma (KIRP; FC, 4.47), ovarian serous cystadenocarcinoma (OV; FC, 6.10), pancreatic adenocarcinoma (PAAD; FC, 21.77), and testicular germ cell tumors (TGCT; FC, 2.99), while the results showed the opposite phenomenon in adrenocortical carcinoma (ACC; FC, 0.27), bladder urothelial carcinoma (BLCA; FC, 0.43), colon adenocarcinoma (COAD; FC, 0.34), acute myeloid leukemia (LAML; FC, 0.13), liver hepatocellular carcinoma (LIHC; FC, 0.18), rectum adenocarcinoma (READ; FC, 0.32), and thymoma (THYM; FC, 0.39) (Figure 4A). We further analyzed the correlation between the transcripts of *FCGR2B* and the HR of patient survival across cancers using univariate analysis. In tumors with an HR score greater than 1, only GBM and KIRC showed significant differences (Figure 4B). Both gene expression and mutations can affect tumorigenesis. However, in our analysis, neither tumor mutational burden (TMB) nor microsatellite instability (MSI) showed a significant relationship with mRNA expression of *FCGR2B* in the GBM database from TCGA (Figures 4C,D). Collectively, these data indicate that FcγRIIb expression is highly related to GBM.

### FcγRIIb is mostly expressed in macrophages and dendritic cells in GBM

**FIGURE 5:**
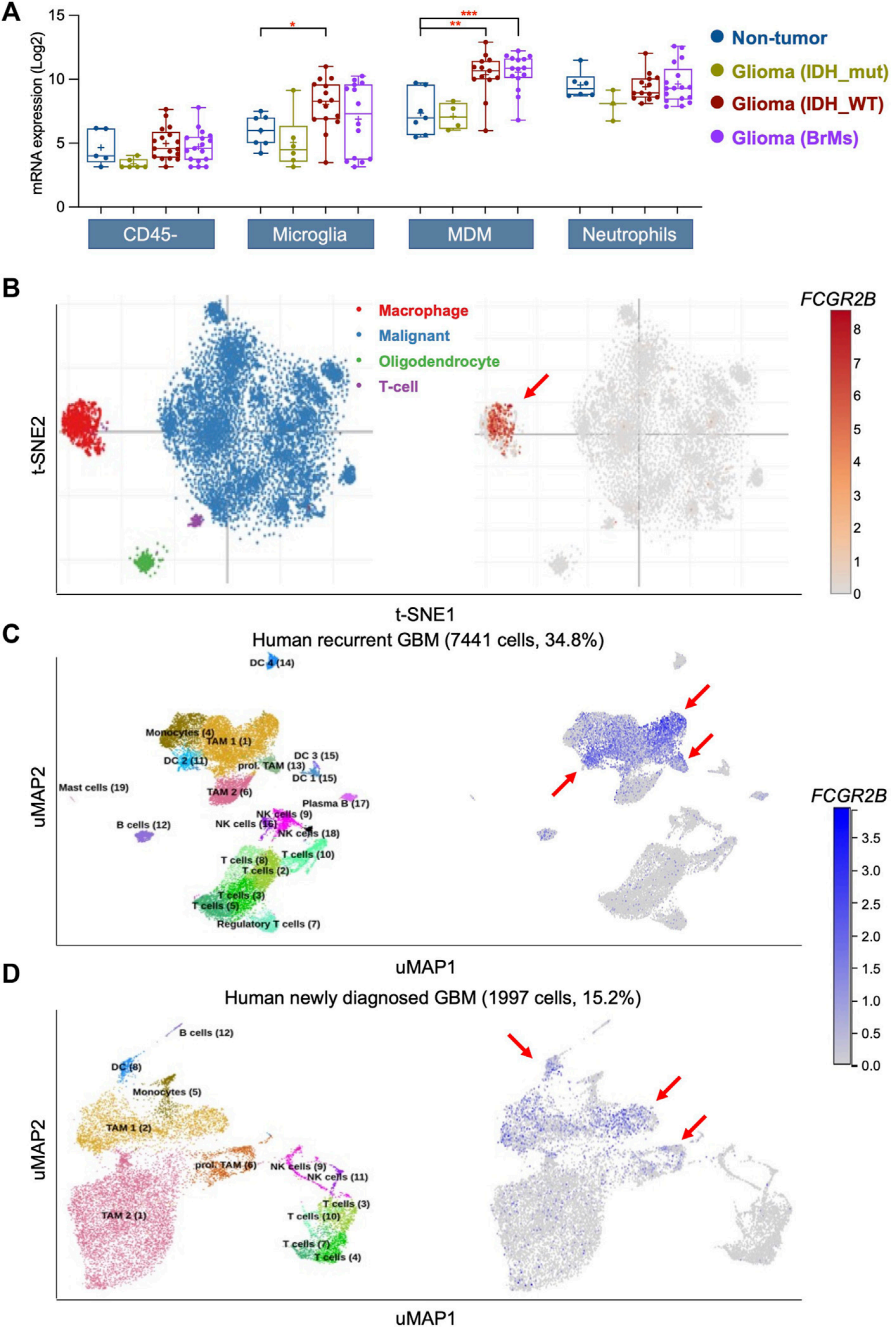
FcγRIIb is mostly expressed in macrophages and dendritic cells in GBM. **(A)** Box plots showing the mRNA expression of *FCGR2B* in CD45^-^ tumor cells, microglia, monocyte-derived macrophages (MDM), and neutrophils in non-tumor, IDH mutant (IDH_mut) type glioma, IDH wild-type (IDH_WT) type glioma, and brain metastases (BrMs) of glioma tissues. *p < 0.05, **p < 0.01, ***p < 0.001. **(B)** The t-distributed stochastic neighbor embedding (t-SNE) clustering of single cells with GBM tissues showing the mRNA expression of *FCGR2B*. Red arrow, main cluster for *FCGR2B*-expressing cells. **(C**,**D)** Uniform Manifold Approximation and Projection (UMAP) clustering of single cells with human recurrent GBM **(C)** and human newly diagnosed GBM **(D)** showing the mRNA expression of *FCGR2B*. Red arrow, main cluster for *FCGR2B*-expressing cells.

Upregulated DEGs from recurrent GBM exhibited a strong enrichment of immune-related signaling pathways (Figures 1B–D). FcγRIIb is one of the most well-known regulators in immune and inflammatory responses. Thus, we hypothesized that FcγRIIb is involved in immune-related signaling pathways or inflammatory cells in GBM tissues. First, through the Brain Tumor Immune Micro Environment (Brain TIME) analysis, we found that *FCGR2B* in microglia and infiltrating monocyte-derived macrophages (MDMs) was more highly expressed in IDH wild-type (IDH_WT) glioma tissues or brain metastases (BrMs) than in non-tumor tissues (Figure 5A). Additionally, the expression of *FCGR2B* was highest in malignant gliomas (IDH_WT and BrMs) in MDMs. Interestingly, despite higher expression levels of all tissue types in neutrophils than in CD45-tumor cells, there were no significant differences between gliomas and non-tumor tissues. Next, we further investigated the cell-type specificity of *FCGR2B* using two kinds of single-cell RNA-Seq databases. Macrophage-specific expression of *FCGR2B* was determined using the Single Cell Portal (Figure 5B). In the other single-cell RNA-seq analysis using the Brain Immune Atlas, we found that *FCGR2B* was almost expressed in tumor-associated macrophages (TAMs) and dendritic cells (DCs) and was expressed at higher levels in recurrent GBM samples than in newly diagnosed samples (Figures 5C, D). Taken together, these data indicate that macrophage- or dendritic cell-derived FcγRIIb is particularly associated with the aggressiveness of recurrent GBM patients and may serve as a therapeutic target for recurrent GBM.

### FcγRIIb regulates immune-associated signaling pathways in recurrent GBM

**FIGURE 6:**
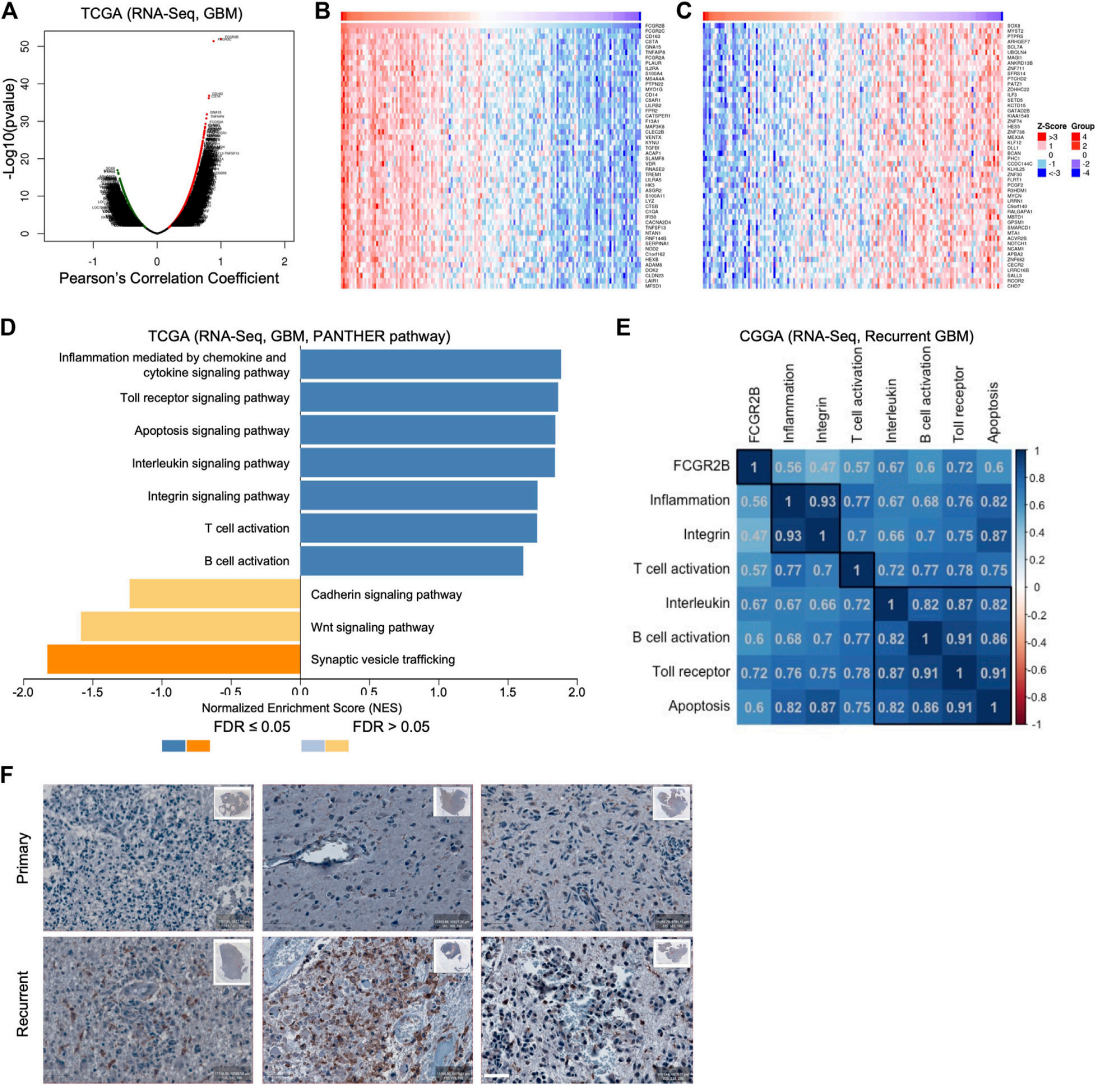
FcγRIIb regulates immune-associated signaling pathways in recurrent GBM. **(A)** Volcano plot showing *FCGR2B*-associated genes from the TCGA cohort ranked by their Pearson’s correlation coefficient R-value and *p*-value. Red dots, positively correlated significant genes, R > 0, *p* < 0.05; black dots, no relationship genes, *p* > 0.05; green dots, negatively correlated significant genes, R < 0, *p* < 0.05. **(B**,**C)** The heatmap shows the top 50 genes that were positively **(B)** and negatively **(C)** correlated with *FCGR2B* expression in the TCGA cohort. **(D)** Bar charts showing the signaling pathways that were significantly enriched in the *FCGR2B* high group (blue bar) and in the *FCGR2B* low group (orange) from the TCGA cohort using GSEA with the Protein ANalysis THrough Evolutionary Relationships (PANTHER) pathway signatures. **(E)** The correlation matrix showing the Pearson’s correlation coefficient between mRNA expression of *FCGR2B* and the enrichment score of immune-associated signaling pathways calculated through ssGSEA using the CGGA recurrent GBM cohort. **(F)** Representative images of immunohistochemistry (IHC) showing FcγRIIb-positive cell populations in primary and paired recurrent specimens from three GBM patients. Scale bar = 50 µm.

To identify the downstream signaling pathway of FcγRIIb, we first analyzed the Pearson’s correlation coefficient between *FCGR2B* and all other genes in the TCGA GBM cohort (Figure 6A). The heatmap shows the mRNA expression of the top 50 genes with positive correlation coefficient values (Figure 6B) and negative correlation coefficient values (Figure 6C) with *FCGR2B*. Interestingly, most of the top 50 genes with positive correlation coefficient values with *FCGR2B* belonged to immune-associated signaling pathways, including *CD14*, *ADAM8*, *TREM1*, *LYZ*, *IL2RA*, and *CD163*. Moreover, we found that immune-associated signaling pathways, such as inflammation mediated by chemokine and cytokine signaling pathway, interleukin signaling pathway, T-cell activation, and B-cell activation, were highly enriched in the *FCGR2B* high group, while synaptic vesicle trafficking, Wnt signaling pathway, and cadherin signaling pathway were enriched in the *FCGR2B* low group through gene set enrichment analysis (GSEA) using the Protein ANalysis THrough Evolutionary Relationships (PANTHER) pathway signatures (Figure 6D). To validate the results, we further analyzed the Pearson’s correlation coefficient between the mRNA expression of *FCGR2B* and the enrichment score of the immune-associated signaling pathways in Figure 6D through single sample gene set enrichment analysis (ssGSEA) using the CGGA cohort. We discovered that all immune-associated signaling pathways showed a very high correlation coefficient with the mRNA expression of *FCGR2B* in recurrent GBM (Figure 6E). Furthermore, we confirmed that FcγRIIb-positive cell populations were markedly higher in recurrent GBM specimens than in primary GBM (Figure 6F, Supplementary Figure S1). Collectively, these results indicate that FcγRIIb regulates immune-associated signaling pathways and provides a promising strategy for immunotherapy in the treatment of recurrent GBM.

## Discussion

In this study, we report FcγRIIb as a unique key modulator of recurrent GBM among various candidates. FcγRIIb was identified by scRNA-Seq analysis to be predominantly expressed in TAMs and DCs in recurrent GBM. With GSEA, we found that FcγRIIb may play an important role in regulating the Toll receptor signaling pathway. Furthermore, FcγRIIb mRNA expression showed a negative correlation with the survival time of patients who harbored recurrent GBM but not primary GBM.

Recent studies have mostly focused on the investigation of differences between primary and recurrent GBM through multiomics analysis. The Glioma Longitudinal AnalySiS (GLASS) consortium employed scRNA-Seq, DNA sequencing, and multiplex immunofluorescence to provide evidence of a correlation between GBM recurrence and IDH mutation status (Varn et al., 2022). They also suggested that IDH mutation status could influence the changes in the GBM subtype, such as neuronal, mesenchymal, and proliferative (Wang et al., 2017), and the microenvironment from an initial tumor to a recurrent one. Another study performed scRNA-Seq and Cellular Indexing of Transcriptomes and Epitopes by Sequencing (CITE-Seq) to illustrate the immune landscape using samples from patients and mice with newly diagnosed GBM and recurrent GBM, and the results revealed a large difference in myeloid compartments, including TAMs, DCs, natural killer (NK) cells, CD8^+^ T cells, and B cells (Pombo Antunes et al., 2021). The results of our DEG and GO analyses using the CGGA cohort were consistent with these reports of significant enrichment of inflammatory signaling pathways in recurrent GBM. Moreover, we demonstrated by scRNA-Seq and GSEA using public GBM scRNA-Seq databases that FcγRIIb, which is expressed on TAMs and DCs, may drive the aggressiveness of recurrent GBM.

Although in our *in silico* analysis, *FCGR2B* was largely expressed in TAMs and DCs in recurrent GBM, it was also expressed in a few T-cells and malignant cells. A recent study has reported that tumor-expressed FcγRIIb could confer the drug-resistance to patients who had diffuse large B-cell lymphoma in the treatment of immunochemotherapy (rituximab plus cyclophosphamide, doxorubicin, vincristine, and prednisone), and it was also correlated with poor prognosis of those patients (Nowicka et al., 2021). In another study of the melanoma model, the authors found that FcγRIIb was upregulated in tumor-infiltrating CD8^+^ T-cells. When the authors genetically eliminated the *FCGR2B*, tumor-infiltrating effector CD8^+^ T cell response was increased and the tumor was decreased (Farley et al., 2021). However, FcγRIIb played a pivotal role in tumor cells or T-cells in those studies based on its high expression level, and whether it is a critical factor in GBM with its few populations in malignant cells and T-cells need more research to prove.

Since the first humanized monoclonal antibody (mAb), trastuzumab, was approved by the Food and Drug Administration (FDA) for the treatment of human epidermal growth factor receptor 2 (HER2)-positive breast cancer, an increasing number of patients have received mAb therapy and prolonged their survival (Adams and Weiner, 2005). Treatment with mAbs shows two kinds of modes of action in patients: immune-independent and immune-dependent mechanisms. The immune-independent mechanism represents the direct binding of the mAb to its target receptor and results in anti-survival consequences, while the immune-dependent mechanism represents the engagement of mAb with FcRs in immune cells (Slamon et al., 2011). Similar to trastuzumab as the best therapeutic option for HER2-positive breast cancer, bevacizumab is used for the treatment of recurrent GBM after radiotherapy. However, FcγRIIb suppresses the antitumor activity of some therapeutic mAbs *in vivo*, including rituximab and trastuzumab (Clynes et al., 2000; Barnhart and Quigley, 2017). This could be explained by the fact that a high expression level of FcγRIIb in recurrent GBM is the major cause of bevacizumab resistance and recurrence.

## Conclusion

Taken together, high expression of FcγRIIb on TAMs and DCs may serve as a potential oncogenic driver through regulating the multiple immune-associated signaling pathways in recurrent GBM, indicating a promising therapeutic strategy of targeting FcγRIIb and the possibility of providing more treatment options to patients with recurrent GBM.

## Data Availability

The original contributions presented in the study are included in the article/Supplementary Material, further inquiries can be directed to the corresponding authors.
