# Supplementary material for: Fc gamma receptor IIb in tumor-associated macrophages and dendritic cells drives poor prognosis of recurrent glioblastoma through immune-associated signaling pathways
Source: Front Genet. 2023 Jan 6;13:1046008. doi: 10.3389/fgene.2022.1046008 (PMC9858204; doi:10.3389/fgene.2022.1046008)
Supplement: Supplementary file 1 [file Image1.pdf]

Supplementary Figure 1.

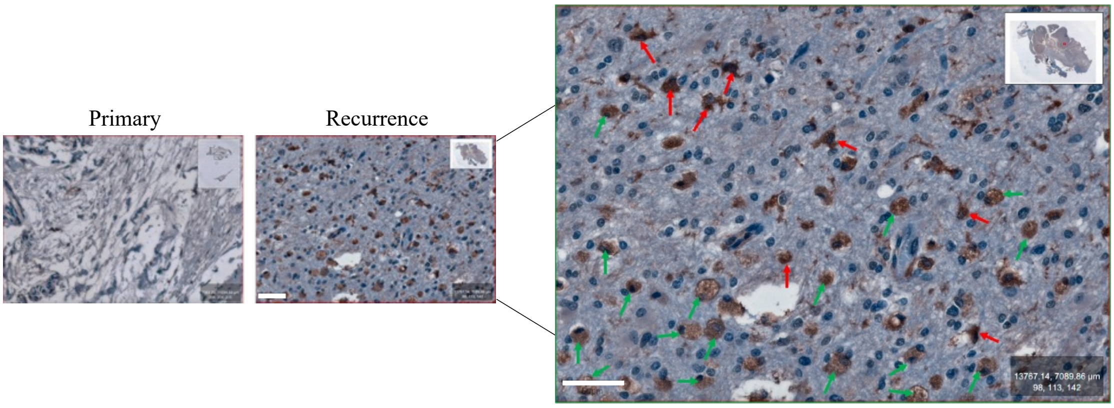

Supplementary Figure 1. Representative images of IHC showing FcγRIIb-positive cell populations in GBM samples. Red arrows, dendritic cells; green arrows, macrophages. The FcγRIIb-positive macrophages and dendritic cells were confirmed by two individual pathologists from Henan Cancer Hospital and Huaihe Hospital of Henan University, respectively. Scale bar = 50 μm.
